# Time trends and sex differences in associations between socioeconomic status indicators and overweight-obesity in Mexico (2006–2012)

**DOI:** 10.1186/s12889-015-2608-2

**Published:** 2015-12-16

**Authors:** Amado D. Quezada, Ana L. Lozada-Tequeanes

**Affiliations:** Nutrition and Health Research Center, Av. Universidad 655 Col. Sta. María, Av. Universidad 655 Col. Sta. María Ahuacatitlán, 62100 Cuernavaca, Morelos Mexico

**Keywords:** Overweight, Obesity, Mexican adults, Sex, Socioeconomic status, Education, Wealth, Marital status, Development

## Abstract

**Background:**

Although the associations between specific socioeconomic status (SES) indicators and overweight or obesity (OWOB) have been studied in different countries, fewer evidence exists for these associations when multiple SES indicators are considered simultaneously. Furthermore, there are few studies investigating time trends in OWOB and their relation with SES in upper-middle income countries, especially for men. The present study contributes to a better understanding of the nature and evolution of the associations between SES indicators and OWOB in the Mexican adult population.

**Methods:**

We pooled data from the 2006 and 2012 National Health and Nutrition Surveys in Mexico and obtained covariate-adjusted prevalence from a design-based logistic multiple regression model. Covariates included a wealth index, education, occupational status, marital status, and all interactions for each covariate with sex (male/female) and survey year.

**Results:**

For men, the association between wealth and OWOB remained positive in general but curvature was more evident in 2012. The wealth-OWOB association in women showed an inverted-U pattern at both years with a positive slope that turned into a negative one as wealth increased. Among women, OWOB prevalence at the college/university education level was approximately 12.0 ± 2.4 (percentage points ± standard error) lower compared with the elementary education level. We did not find differences between educational categories for men in 2006, but in 2012 OWOB tended to be higher among the more educated. The prevalence of obesity in women increased at wealth levels from the middle and upper-middle section of the wealth distributions. Overall OWOB prevalence was near 70 % in 2012 for both sexes.

**Conclusions:**

Among Mexican women, the associations between SES indicators and excess body weight were consistent to those found in developed countries. Among Mexican men, higher education was not associated with a lower prevalence of OWOB but the positive association between wealth and OWOB weakened as wealth increased. The overall prevalence of OWOB was very high for both sexes; its reduction should remain a public health priority given the consequences of nutrition-related chronic diseases, disability and health care costs.

**Electronic supplementary material:**

The online version of this article (doi:10.1186/s12889-015-2608-2) contains supplementary material, which is available to authorized users.

## Background

The rise of obesity is an important public health concern due to its impact on nutrition-related chronic diseases such as diabetes mellitus, cardiovascular disease, and cancer, and its toll on disability and health care costs [1, 2]. High body mass index (BMI) is the second most important risk factor for death and disease burden in Mexico [3]. The combined prevalence of overweight and obesity (OWOB) in Mexican adults has increased considerably over the past two decades. For women between 20 and 49 years of age it rose from 34.5 % in 1988 to 71.4 % in 2006 and to 70.5 % in 2012. For men older than 20 years of age, OWOB increased from 60.7 % in 2000 to 66.7 % in 2006 and to approximately 70.0 % in 2012 [4]. Mexico is now second, after the United States, among the OECD [5] countries with the highest adult obesity prevalence, and has been classified among the top 20 countries with the highest prevalence of adult OWOB in the world [6]. Although overall changes from 2006 to 2012 may not seem substantial, it is important to investigate if there have been significant changes in the distribution of OWOB among subpopulation groups in order to better understand the epidemic and who it is affecting most.

Socioeconomic indicators such as wealth, education, occupation, and marital status have been associated with OWOB or obesity in previous studies [7–9]. These factors may affect bodyweight through influences on physical activity and diet [10, 11]. In developed countries, higher socioeconomic status (SES) has been associated with decreased obesity risk, especially among women. In contrast, among developing countries, higher SES has been associated with an increased risk of obesity [7, 12]. Sex-specific associations between socioeconomic indicators and OWOB have been extensively studied in high income countries, but fewer studies have investigated those associations in middle income countries [13], with even fewer [14] addressing time trends for these specific associations in upper-middle income countries such as Mexico.

Although socioeconomic indicators are correlated with each other (e.g. education and wealth), they may also be reflecting some specific relationships with OWOB that emerge when analyzed simultaneously. Therefore, it may be useful to estimate associations of each indicator when controlling for other indicators. For example, if two socioeconomic indicators are correlated positively with each other and also with OWOB but one of the indicators is omitted from the analysis, the expected value of the association of the included SES indicator with OWOB would be biased upwards. On the other hand, if the two SES indicators have opposite associations with OWOB and the association between the included SES indicator and OWOB is positive, the expected value of the estimated association would be biased downwards (given a positive correlation between SES covariates).

To the best of our knowledge, this is the first study addressing associations between multiple socioeconomic indicators, and OWOB for each sex in the Mexican adult population. Additionally, this is the first study estimating OWOB trends at different wealth levels with the most recent nationally representative surveys in Mexico. Knowing how associations have evolved during this study period may be helpful for determining whether the epidemic concentrates in certain characteristics of the population, and if Mexico then needs to refocus policies aimed towards reducing OWOB.

## Methods

### Data

The Mexican National Health and Nutrition Survey (NHNS) was administered to a multi-stage stratified cluster sample in 2006 and 2012. Each sample was representative of rural (<2500 inhabitants) and urban (≥2500 inhabitants) areas in each Mexican federal state. The ethics review board from the National Institute of Public Health in Mexico approved the study protocol, and all participants provided informed consent.

### Variables and sample

The adult (20 years or older) anthropometric sample from 2006 included 13,358 men and 20,426 women and the 2012 adult anthropometric sample included 15,997 men and 22,231 women. The two design-based cross sectional samples were pooled. Subjects older than 75 years of age and pregnant women were excluded (5.9 % of the pooled sample). Body height and body weight were measured using standard procedures [15, 16]. BMI was calculated as weight in kilograms divided by height in meters squared. BMI values less than 10 kg/m2, or greater than 58 kg/m2, were considered invalid (0.5 % of the pooled sample). OWOB was defined as BMI ≥ 25 kg/m2 and obesity as BMI ≥ 30 kg/m2.

Weights for the construction of a wealth index were obtained from the 2006 data extracting the first principal component; items included household material characteristics, source of household water, electricity, and possession of durable goods. The obtained weights were then applied to the 2012 data and scores for the pooled sample standardized with respect to 2006; that is, in 2006 the wealth index has zero mean and unit variance. More details on the construction of the wealth index are presented in Additional file 1.

Level of education was determined by the last school year completed, and were categorized into five levels: no formal education, elementary school, middle school, high school, college/university or higher. In Mexico, elementary school comprises first through sixth grades, middle school comprises seventh through ninth grades, and high school comprises tenth through twelfth grades. We grouped technical training after high school with the college/university category. We categorized marital status into three groups: single, married/cohabitating, and divorced/widow and occupational status into four groups: student, housekeeper, paid worker and other. The “other” category included the retired, the disabled and those who work in a family business without pay. States were grouped into four regions: north, central, Mexico City, and south. Methodological details about the NHNS 2006 and 2012 have been published previously [4]. A small proportion of the pooled sample (0.2 %) with “don’t know/no answer” response for education and marital status were eliminated since this response option was only available in 2006.

### Statistical analysis

We fitted two multiple logistic regression models to the pooled sample, one with OWOB and the other with obesity as outcome variable. The same specification for the linear predictor was used for both models. Age and the wealth index were specified as continuous covariates including their squared terms. Sex, survey year, education, marital status, occupational status, and area of residence were specified as categorical covariates. We included all interactions up to third-order for each covariate with sex and survey year in our model. In this way, we could obtain sex-year specific estimates from the same model, and perform comparison tests between sex-year groups. Survey design was taken into account in estimation and standard errors obtained through linearization [17]. For each sex and survey year, covariate-adjusted prevalence were estimated through predictive margins [18]. More details on the estimation and interpretation of predictive margins are provided in Additional file 2. We compared these adjusted prevalence between categories of each covariate and estimated differences between sexes and survey years. We obtained predictive margins at given wealth levels and estimated their covariate-adjusted slopes with average marginal effects [19]. Since area of residence has been suggested to act as a moderator variable [20], we estimated a model with interactions between area of residence and all covariates and performed joint significance tests for each sex-year on each group of SES indicators. Given the exploratory nature of this research, we presented results by area of residence whenever the group of interactions was significant at the 0.1 level. Results and more details on these interactions are available as Additional file 3

Additionally, we estimated models with wealth as categorical using eight equally spaced categories (Additional file 4), and found a similar pattern to that described by the quadratic functional form. We set the threshold for statistical significance at 0.05 for all prevalence comparisons.

In order to assess changes in standard errors when adjusting for both education and wealth, we compared results from three model specifications: Model 3 corresponds to the main model described before, Model 1 includes all covariates from Model 3 except all terms related to the wealth index, and Model 2 includes all covariates from Model 3 except all terms related to education level. We compared odds ratios between the three models for each sex and year combination.

All analyses were performed in Stata v.12.1 [21]. Graphical displays of the associations of OWOB or obesity with the wealth index were produced in R v. 3.2.1 [22]. We defined the range of wealth values for such plots by the overlapping of 5^th^ percentile to 95^th^ percentile intervals between survey years. Details on such ranges are available in Additional file 1.

## Results

### Sample size and descriptive statistics

The analytic sample is composed of adult subjects 20 to 75 years old with an average age of about 41 years (Table 1), with approximately 20 % from rural areas. The wealth index, which was standardized with respect to 2006, ranged from −3.8 to 1.5 standard deviations (SD). From 2006 to 2012, the mean of wealth increased about 0.1 SD. The percentage of subjects with middle school education or higher also increased from 2006 to 2012. The majority of subjects were married or lived with their partners. About 3 % of subjects identified themselves as students, approximately 83 % of men and one third of women identified themselves as paid workers and about 1 % of men and 60 % of women identified themselves as housekeepers. Approximately 70 % of men and 74 % of women were OWOB in 2012.

**Table 1:** Sample size and survey-weighted descriptives of analytical sample

	Males		Females	
	2006	2012	2006	2012
Sample size	12,520	15,140	18,938	20,711
Expanded sample (thousands)	23,025	31,205	32,319	34,425
Age in years (mean ± SD)	41.8 ± 14.7	40.7 ± 13.6	41.2 ± 14.6	41.2 ± 14.9
Standardized^a^ Wealth Index				
(mean ± SD)	0.0 ± 1.0	0.1 ± 0.9	0.0 ± 1.0	0.1 ± 1.0
Median [p25, p75]	0.2 [−0.4, 0.8]	0.3 [−0.4, 0.9]	0.1 [−0.5, 0.7]	0.3 [−0.4, 0.9]
Education level				
No education (%)	7.4	5.4	10.2	7.4
Elementary school (%)	37.7	30.6	43.1	34.6
Middle school (%)	24.8	30.4	21.7	29.4
High school (%)	15.3	17.9	14.4	15.9
College/University (%)	14.7	15.7	10.6	12.7
Marital Status				
Single (%)	21.0	22.6	18.1	18.3
Married/cohabitating (%)	74.2	72.4	68.1	66.0
Widowed/separated (%)	4.8	5.0	13.8	15.7
Occupational status				
Other^b^ (%)	13.0	12.5	6.9	3.8
Student (%)	2.8	3.4	2.2	2.8
Housekeeper (%)	0.8	1.3	60.3	56.9
Paid worker (%)	83.4	82.8	30.6	36.5
Contry region				
North (%)	21.6	21.1	19.5	19.6
Centre (%)	28.9	28.9	30.1	29.0
Mexico City (%)	20.3	19.8	20.4	20.3
South (%)	29.2	30.2	29.9	31.1
Area of residence				
Urban (%)	80.3	78.3	79.4	79.3
Rural (%)	19.8	21.7	20.6	20.7
Raw prevalence				
BMI ≥ 25 (%)	67.6	70.1	72.8	73.7
BMI ≥ 30 (%)	24.8	27.4	35.2	38.1

^a^ Wealth Index obtained by extracting the first principal component from household material characteristics, source of household water, electricity and possession of durable goods, and standardized with respect to 2006

^b^Includes the retired, the disabled and workers in family business without pay

The distribution of the unweighted sample over the socioeconomic covariate categories (not shown) was very similar to that presented in this table

### Overweight plus obesity and socioeconomic status

The covariate-adjusted prevalence of OWOB from the multiple logistic regression model are presented in Table 2. In 2006, among men there were no statistically significant differences between OWOB and education levels. However, in 2012, men with high school or college/university education had a prevalence of OWOB about 5.0 ± 2.2 percentage points (p.p. ± standard error) higher than those with elementary education. In contrast, among women at the elementary school level or higher, education was associated with a decreasing prevalence of OWOB; in both survey years, women with college/university education had a prevalence of OWOB about 12.0 ± 2.4 p.p. lower than women with only an elementary education. In men, there was a significant increase in OWOB from 2006 to 2012 at the no education (8.0 ± 4.0 p.p.), high school (8.5 ± 3.4 p.p.) and college/university (9.8 ± 4.5 p.p.) categories.

**Table 2:** Covariate-adjusted prevalences of overweight plus obesity (BMI ≥ 25)

	Men		Women	
	2006	2012	2006	2012
Education level				
No education	63.4^a^ ± 3.2	71.4^abc^* ± 2.4	74.1^ab#^ ± 1.7	70.8^a^ ± 1.8
Elementary school	66.8^a^ ± 2.4	71.0^a^ ± 1.6	76.0^a#^ ± 0.9	77.3^b#^ ± 1.0
Middle school	66.9^a^ ± 2.6	72.0^ac^ ± 1.6	73.0^bc#^ ± 1.2	74.9^c^ ± 0.9
High school	67.0^a^ ± 2.9	75.5^b^* ± 1.7	69.7^c^ ± 1.4	70.7^a#^ ± 1.3
College/University	66.2^a^ ± 4.0	76.0^bc^* ± 1.9	64.4^d^ ± 2.3	64.8^d#^ ± 1.8
Marital status				
Single	58.9^a^ ± 2.8	64.7^a^ ± 2.0	68.6^a#^ ± 1.4	68.7^a^ ± 1.3
Married/cohabitating	69.5^b^ ± 2.4	75.7^b^* ± 1.3	73.8^b^ ± 0.9	74.5^b^ ± 0.7
Widowed/separated	62.0^ab^ ± 4.8	70.6^c^ ± 2.7	71.5^ab^ ± 1.4	76.0^b^* ± 1.4
Occupational status				
Other^†^	68.9^a^ ± 1.9	67.3^a^ ± 1.7	70.2^a^ ± 2.2	73.9^a#^ ± 2.6
Student	57.7^a^ ± 5.9	68.9^ab^ ± 3.8	64.5^a^ ± 4.6	71.7^a^ ± 3.3
Hosekeeper	64.6^a^ ± 6.9	78.5^b^ ± 3.6	72.0^a^ ± 0.9	72.6^a^ ± 0.7
Paid worker	67.8^a^ ± 1.0	70.4^a^* ± 0.7	73.4^a#^ ± 1.0	73.7^a#^ ± 0.8
Overall prevalence	66.5 ± 2.3	72.7* ± 1.2	72.4^#^ ± 0.7	73.3 ± 0.6

All estimates are covariate-adjusted prevalence ± standard errors obtained through predictive margins from a survey design-based multiple logistic regression model. Country region, area of residence, a wealth index and its squared term, age and age squared were included in the model along with the other covariates

^†^Includes the retired, the disabled and workers in a family business without pay

Different letters indicate significant differences (*P* < 0.05) between covariate categories in the same sex and survey year

*P < 0.05 change from 2006 to 2012 within sex

^#^ *P* < 0.05 men vs. women at the same survey year

In both survey years the prevalence of OWOB was approximately 11.0 ± 1.9 p.p. higher for married/cohabitating men than for single men and 5.5 ± 1.6 p.p. higher for married/cohabitating women than for single woman. In men, OWOB significantly increased at the married/cohabitating and paid worker categories.

**Fig. 1:**
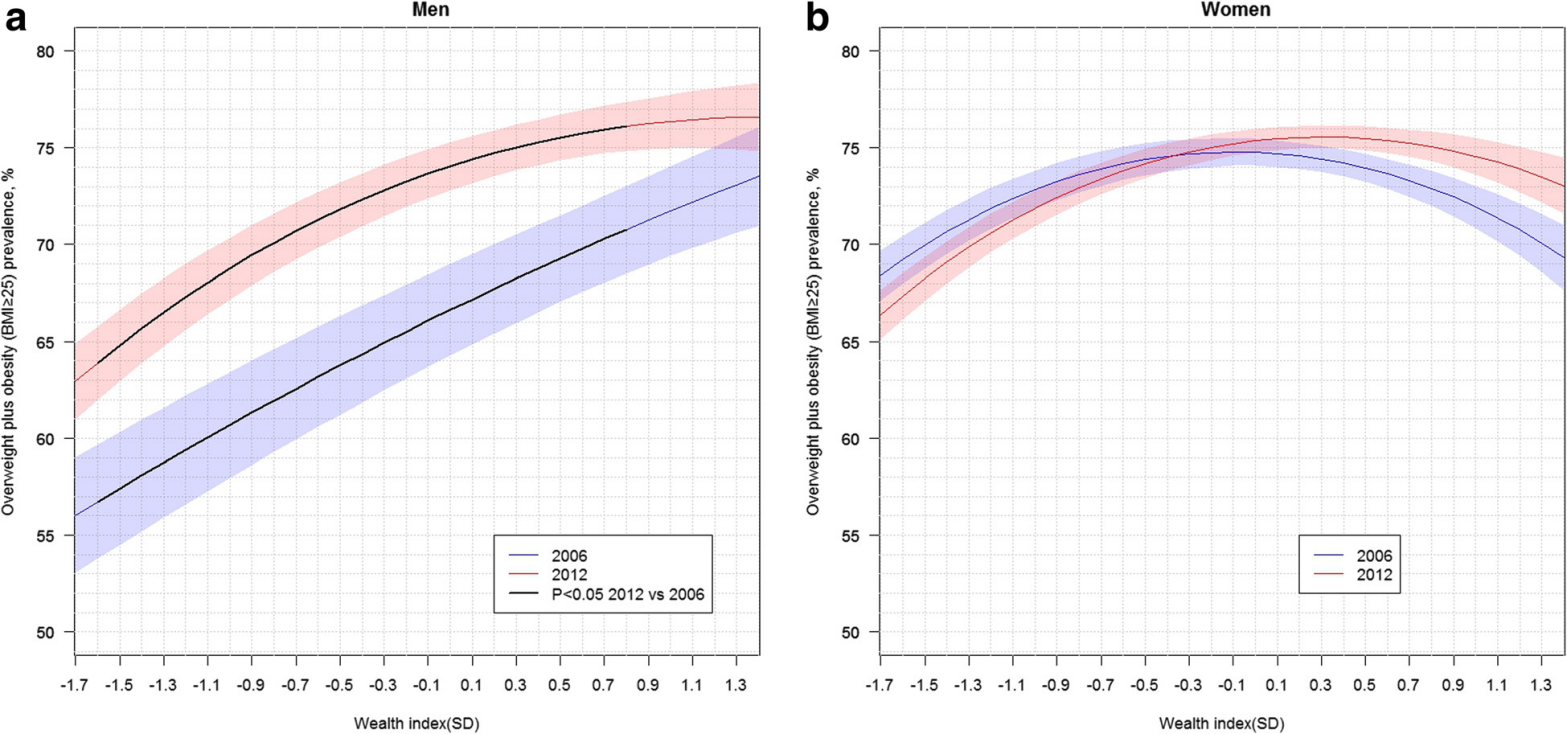
Covariate adjusted wealth index associations with overweight plus obesity (BMI ≥ 25), by survey year, for men (panel **a**) and women (panel **b**). Covariate adjusted prevalence are presented. Adjustment covariates included age, age squared, education level, marital status, occupational status, country region and area of residence. Wealth Index obtained by extracting the first principal component from household material characteristics, source of household water, electricity and possession of durable goods, and standardized with respect to 2006

In men the covariate-adjusted association between wealth status and OWOB changed from being positive and nearly linear in 2006 to a curved one in 2012 (Fig. 1). The slope in 2012 was positive and considerably steep at low wealth levels but decreased with wealth and vanished at wealth levels above 0.5 SD. Estimated slopes at given wealth levels along with their standard errors are available as Additional file 5. The prevalence of OWOB significantly increased in men at wealth levels from −1.6 to 0.8 SD (Fig. 1), and based on inter-quartile ranges (Table 1) these values corresponded to the low and middle sections of the wealth distributions. In women the association between wealth status and OWOB remained similar from 2006 to 2012 although the maximum of the function slightly shifted to the upper-right. Among women, wealth status was positively associated with OWOB for wealth values located below the median (<−0.3 SD in 2006; <0.1 SD in 2012) and negatively associated with OWOB for wealth values located above the median (>0.2 SD in 2006; >0.7 SD in 2012).

### Obesity and socioeconomic status

**Fig. 2:**
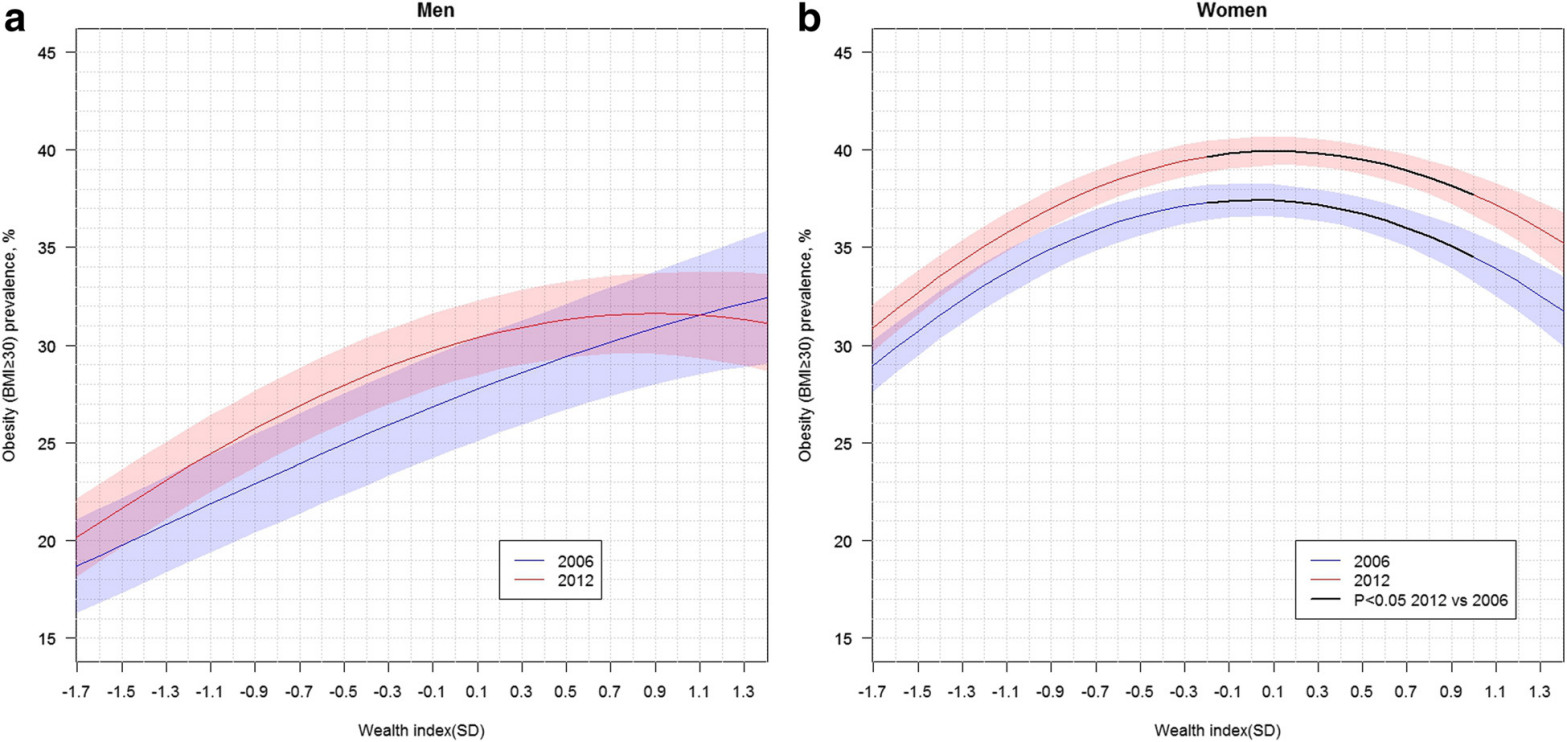
Covariate adjusted wealth index associations with obesity (BMI ≥ 30), by survey year, for men (panel **a**) and women (panel **b**). Covariate adjusted prevalence are presented. Adjustment covariates included age, age squared, education level, marital status, occupational status, country region and area of residence. Wealth Index obtained by extracting the first principal component from household material characteristics, source of household water, electricity and possession of durable goods, and standardized with respect to 2006

Covariate-adjusted prevalence of obesity are presented in Table 3. Women at the college/university level showed much lower obesity prevalence than women with middle-school education or below. The sex-year specific covariate-adjusted associations between wealth status and obesity are shown in Fig. 2. We observed a similar pattern to that described for OWOB. Among women, obesity significantly increased from 2006 to 2012 at wealth values located in the middle and upper-middle of the wealth distributions (> − 0.2 SD and <1.0 SD) but the association between wealth and obesity remained negative for wealth values above the mean in both years. Estimated slopes for the association between wealth and obesity at given wealth levels are available as Additional file 5 for both sexes in 2006 and 2012.

**Table 3:** Covariate adjusted prevalence of obesity (BMI ≥ 30)

	Men		Women	
	2006	2012	2006	2012
Education level				
No education	24.0^a#^ ± 3.2	26.6^a#^ ± 2.7	37.3^a^ ± 2.1	36.8^a^ ± 1.9
Elementary school	28.6^a#^ ± 2.6	27.9^a#^ ± 2.0	38.4^a^ ± 1.2	41.6^b^* ± 1.1
Middle school	27.4^a#^ ± 3.1	29.9^a#^ ± 2.1	35.6^a^ ± 1.4	37.2^a^ ± 1.1
High school	27.2^a^ ± 3.0	29.8^a^ ± 2.4	30.1^b^ ± 1.6	34.3^a^* ± 1.5
College/University	25.1^a^ ± 3.1	29.6^a^ ± 2.4	26.4^b^ ± 1.9	30.0^c^ ± 1.8
Marital status				
Single	21.9^a#^ ± 2.5	26.7^a#^ ± 2.4	32.0^a^ ± 1.7	35.6^a^ ± 1.4
Married/cohabitating	28.0^b#^ ± 2.7	29.8^a#^ ± 1.9	35.4^a^ ± 0.9	37.8^a^* ± 0.8
Widowed/separated	31.1^ab^ ± 5.2	26.8^a#^ ± 2.8	35.3^a^ ± 1.8	37.9^a^ ± 1.3
Occupational status				
Other^†^	28.6^a^ ± 1.9	28.7^a#^ ± 1.7	32.9^a^ ± 2.3	37.0^a^ ± 2.8
Student	28.5^a^ ± 5.5	24.6^a^ ± 4.0	32.3^a^ ± 5.2	35.4^a^ ± 4.5
Housekeeper	31.5^a^ ± 7.5	32.7^a^ ± 5.5	34.6^a^ ± 0.8	38.3^a^* ± 0.8
Paid worker	24.6^a#^ ± 0.9	27.0^a#^* ± 0.7	35.3^a^ ± 1.2	37.1^a^ ± 1.0
Overall prevalence	27.3 ± 2.5	28.9 ± 1.8	34.8 ± 0.7	37.4* ± 0.6

All estimates are covariate-adjusted prevalence ± standard errors obtained through predictive margins from a survey design-based multiple logistic regression model. Country region, area of residence, a wealth index and its squared term, age and age squared were included in the model along with the other covariates

^†^Includes the retired, the disabled and workers in a family business without pay

Different letters indicate significant differences (*P* < 0.05) between covariate categories in the same sex and survey year

*P < 0.05 change from 2006 to 2012 within sex

^#^ *P* < 0.05 men vs. women at the same survey year

### Area of residence interactions with SES indicators

Interactions with area of residence were joint-significant (*p* < 0.05) only for the wealth-OWOB association among men in 2012; from 2006 to 2012, OWOB increased mainly in urban areas, and curvature of the 2012 function was more evident for rural areas (Additional file 6). The interactions between area of residence with education in men and marital status in women suggested possible differential relationships (*p* < 0.1). The specific estimates for area of residence and each of the aforementioned covariate categories are available in Additional file 7.

### Comparison of models depending on the inclusion of wealth, education, or both as covariates

We adjusted two additional model specifications (Additional file 8). The first excludes education indicators and leaves wealth terms and the second excludes wealth terms but leaves education indicator variables. Standard errors remained stable when adjusting for both groups of variables. Compared to changes in wealth coefficients, education coefficients changed more markedly when adjusting for both education and wealth. In models that included both groups of covariates, education coefficients tended to be lower than when compared to models that included education but not wealth.

## Discussion

The present study examined associations between multiple SES indicators and OWOB, and their evolution in the Mexican adult population from 2006 to 2012. Associations among women were generally consistent with the body of evidence on the relation between obesity and SES from highly developed countries. For low to middle wealth levels in women and for men in general, results were more concordant with what has been observed in low-middle developed countries [7].

We found that prevalence of obesity among Mexican women increased from 2006 to 2012 at wealth values from the middle to the upper-middle part of the wealth distribution. It has been suggested that the burden of obesity shifts from higher to lower SES as per capita income increases in a given country [13]; from 2006 to 2012 real per capita income increased about 13 % in Mexico [23]. On the other hand, recent research has shown that in developing countries with relatively high per capita income and high-income inequality (Bolivia, Peru, Guatemala, Namibia, and Colombia), the prevalence of OWOB in adult women increased more rapidly in the wealthier groups [24]. However, the authors interpreted these results conservatively; the availability of more data would clarify if this pattern persists. As of 2010, Mexico had a Gini index of 47.2 [25], which would correspond to category of high income-inequality (Gini index from 42.2 to 74.3) reported in the aforementioned study [24]. Although obesity in Mexican women significantly increased from 2006 to 2012 among wealth values located in the middle and upper-middle of the wealth distributions, the association between wealth and obesity remained negative for wealth values above the mean at both survey years.

In various SES categories, the proportion of subjects with no excess body weight (BMI < 25) decreased in men from 2006 to 2012. On the other hand, the proportion of women with no excess body weight did not significantly change. However the distribution of women with OWOB was concentrated at higher BMI values, which resulted in the shift of prevalence from overweight to obesity. This increases the risks of chronic diseases [26–28].

Contrary to the perception that OWOB or obesity is shifting to low SES groups, we found that OWOB increased among men with a high education level, and obesity increased among women with relatively high wealth index, as previously noted. Additional research is required to properly identify the determinants of such increases. One possibility could be higher accessibility to and consumption of ultra-processed foods. Retail sales per-capita of ultra-processed drinks and food products increased 29.2 % from 2000 to 2013 in Mexico, and exposure to ultra-processed food products has been linked to urbanization and a higher per-capita income, among other factors [29]. In regard to formal education and OWOB prevalence among men, our results underscore the necessity to strengthen nutritional and health education within school programs from early grades on up to the highest grades. Prevention at early ages in life should be reinforced. The high prevalence of OWOB in Mexico is justification for public health policy targeted to all population groups, with a focus on subpopulations that are at higher risk of OWOB. In Mexico, public policy is aligning toward OWOB prevention through a combination of interventions in multiple sectors [29, 30].

We found that OWOB prevalence was higher at the married/cohabitating category compared to the single category, with a greater difference for men. Single individuals may have more awareness of body shape and assign it a greater value. The difference between male and female may reflect the fact that women are more aware of body size than men. Even when married, women may still tend to pursue thinness, which is a culturally reinforced value particularly in developed countries [12, 31].

Other factors previously shown to modify weight include accessibility to foods with high caloric density, availability of opportunities to engage in physical activity, and lack of public awareness of overweight health hazards [32, 33]. Individuals with a high SES have more available resources to modify their diet and physical activity and can therefore more easily regulate their weight. It is possible that women become more culturally connected to western body size values as SES increases.

Most studies relating SES to OWOB or obesity are limited to cross sectional samples, even fewer studies have assessed sex-specific change in the prevalence of OWOB or obesity at categories of SES indicators. Most recent studies on women from low and middle-income countries have focused on either wealth or education as a SES measure or analyzed them separately [24, 34]. We used a multivariate approach for estimating associations between various SES indicators, which allowed us to avoid potential biases caused by omitting any of the available SES indicators. Therefore, coefficients our estimates reflect associations attributable to the variable in question when all other covariates are held constant. It is possible that wealth is a mediator between education and OWOB. We did not estimate this potential mediation, but our additional analyses indicated that part of the total relationship between education and OWOB may be mediated by wealth (Additional file 8).

Although each indicator attempts to measure SES, they may be reflecting different aspects of development. The distinction may be especially relevant when a country is at a transitional stage of its development; it could be that as countries develop, these associations converge. Knowing whether individual SES indicators converge or diverge in their associations with excess body weight highlights the importance of a multivariate approach to this analysis. Under isolation from other SES indicators, this approach can identify specific socioeconomic categories more closely related to excess body weight.

Our approach for assessing change in OWOB or obesity at given wealth levels was different from other studies; we could apply the exact same definition of wealth in both years since the wealth items were identical in both surveys. For more time-distant surveys, definitions of some of the items and their meaning as indicators of wealth may change. Under such circumstances using quintiles or other distributional categorization would be preferable.

The present study is based on the 2006 and 2012 NHNS surveys, which are representative of the Mexican population. The most recent studies relating SES to obesity in the Mexican adult population were limited either to the 2006 or to the 2012 data [35, 36], or focused solely on trends in women and educational categories [20].

Limitations of our study should be noted. We did not include dietary intake, physical activity, smoking or parity in the analyses. Both dietary intake and physical activity have been recognized as mediator variables in previous literature [10, 11, 31], and as such their omission would result in the estimation of total associations of SES with excess body weight. That is, the calculated associations may incorporate or absorb the pathway through these mediators.

Interactions for area of residence were significant only for the wealth-OWOB association among men. Graphical analysis showed that wealth ranges have lower levels concentrated in rural areas. This may be driving the interaction significant since area of residence groups do not sufficiently overlap at low wealth levels. On the other hand, standard errors were much larger for the rural area, which also complicates detection of actual changes between 2006 and 2012.

Failure to reject the null of no interactions from our joint tests for area of residence does not imply that such interactions do not exist. Sample sizes of the SES categories within rural areas were relatively small and therefore could result in less precise estimates. Furthermore, given the correlation between the included SES indicators, standard errors are expected to be greater than a situation in which the covariates are not correlated.

## Conclusions

Our results showed that covariate-adjusted prevalence of OWOB and obesity varied different ways depending on sex and type of SES indicator. Among women with some education, the level of education was negatively associated with OWOB and obesity. Women with a relatively high level of wealth also showed a negative association to OWOB. These results were consistent to those found in developed countries. In contrast, among Mexican men, high education was not related with a lower prevalence of OWOB. The positive association between wealth and OWOB became stronger at low wealth levels but vanished at high wealth levels from 2006 to 2012. Our results indicate that obesity is increasing among women with higher wealth levels and OWOB increasing in men in general. Education seems to play a different role between women and men. Further research is needed to determine the underlying forces behind these differences. The overall prevalence of OWOB in 2012 was very high in Mexico. Given the consequences of OWOB on nutrition-related chronic diseases, disability, and health care costs, it should remain a public health priority. Policies aimed to OWOB reduction should be directed to all the population but with special emphasis on prevention and the most vulnerable groups to the epidemic.

## Additional files

Additional file 1:
**Calculation of the wealth index.** (PDF 65 kb) Additional file 2:
**Calculation of predictive margins.** (PDF 88 kb) Additional file 3:
**Interaction tests with area of residence.** (PDF 57 kb) Additional file 4:
**Wealth relationships with OWOB and obesity using equally spaced wealth categories.** (PDF 93 kb) Additional file 5:
**Covariate adjusted slopes for the association between wealth and OWOB and obesity at given wealth levels, by sex and survey year.** (PDF 87 kb) Additional file 6:
**Wealth associations for men, by area of residence.** (JPEG 1072 kb) Additional file 7:
**Estimates from interactions with area of residence.** (PDF 58 kb) Additional file 8:
**Comparison of models depending on the inclusion of wealth, education, or both as covariates.** (PDF 59 kb)
