# Supplementary material for: Time trends and sex differences in associations between socioeconomic status indicators and overweight-obesity in Mexico (2006–2012)
Source: BMC Public Health. 2015 Dec 16;15:1244. doi: 10.1186/s12889-015-2608-2 (PMC4682269; doi:10.1186/s12889-015-2608-2)
Supplement: Additional file 1: — Calculation of the wealth index. (PDF 65 kb) [file 12889_2015_2608_MOESM1_ESM.pdf]

Additional File 1. Calculation of the wealth index

Weights for the construction of a wealth index were obtained from the 2006 data extracting the first principal component. The obtained weights were then applied to the 2012 data and scores for the pooled sample standardized with respect to 2006, that is, in 2006 the wealth index has zero mean and unit variance.

Table AF1-1. Correlations of items with first principal component and scoring coefficients

Item	Correlations with First Principal Component	Scoring coefficients*
Household materials and services		
Floor charactesitics	0.654	0.153
Ceiling characteristics	0.511	0.120
Water facilities	0.568	0.133
Electricity	0.277	0.065
Posession of durable goods		
Car	0.490	0.115
Wagon	0.203	0.048
Audio system	0.562	0.132
Refrigerator	0.677	0.159
Gas stove	0.662	0.155
Boiler	0.635	0.149
Computer	0.489	0.115
Microwave	0.606	0.142
Blender	0.614	0.144
Television	0.546	0.128

The first principal component absorbed 30% (4.27/14) of total variation in 2006
*Obtained with the regression method

Table AF1-2. Survey-weighted descriptive statistics of wealth index by survey year

Year	Percentiles					Mean	Standard Deviation
	p5	p25	p50	p75	p95		
2006	-2.1	-0.5	0.1	0.7	1.4	0.0	1.0
2012	-1.7	-0.4	0.3	0.9	1.4	0.1	0.9

The region of wealth index values defined by overlapping the (p5, p95) intervals between survey years was (-1.7, 1.4)

Table AF1-3. Survey-weighted descriptive statistics of wealth index by area of residence and survey year

Year	Percentiles					Mean	Standard Deviation
	p5	p25	p50	p75	p95		
Urban area							
2006	-1.2	-0.2	0.3	0.9	1.4	0.3	0.8
2012	-1.1	-0.2	0.4	0.9	1.4	0.3	0.8
Rural area							
2006	-3.0	-1.8	-0.8	-0.2	0.6	-1.0	1.1
2012	-2.5	-1.3	-0.5	0.1	0.9	-0.6	1.0

In the urban area the region of wealth index values defined by overlapping the (p5, p95) intervals between survey years was (-1.1, 1.4)

In the rural area the region of wealth index values defined by overlapping the (p5, p95) intervals between survey years was (-2.5, 0.6)
