# Supplementary material for: Time trends and sex differences in associations between socioeconomic status indicators and overweight-obesity in Mexico (2006–2012)
Source: BMC Public Health. 2015 Dec 16;15:1244. doi: 10.1186/s12889-015-2608-2 (PMC4682269; doi:10.1186/s12889-015-2608-2)
Supplement: Additional file 2: — Calculation of predictive margins. (PDF 88 kb) [file 12889_2015_2608_MOESM2_ESM.pdf]

Additional File 2. Calculation of predictive margins

Predictive margins can be used for obtaining covariate-adjusted means from a generalized linear model. We specified the logit as a link function between the linear predictor $\mathbf{x}_i'\widehat{\boldsymbol{\beta}}$ and the mean $\hat{\mu}_i$ (Equation 1).

Equation 1 $\ln\left(\frac{\hat{\mu}_i}{1-\hat{\mu}_i}\right) = \mathbf{x}_i'\widehat{\boldsymbol{\beta}}$ $\mathbf{x}_i$: vector of covariates; $\boldsymbol{\beta}$: vector of model parameters.

Given that our response variable is binary, the conditional mean on the covariates corresponds to a conditional probability; furthermore, since we used a logit link function, adjusted means or probabilities are constrained to the 0 to 1 scale.

It has been common practice in applied research to obtain covariate adjusted probabilities by setting adjustment covariates at their sample mean and then applying the inverse of the link function to the linear predictor. In contrast, a predictive margin is the mean of predicted probabilities. Instead of setting adjustment covariates at their sample mean and calculating probabilities at this single point, probabilities are predicted for each individual and then averaged over the sample. We will illustrate this with a very simple model. Suppose that the probability of overweight or obesity (BMI≥25) is modelled to depend on age (years), a wealth index (SD), sex (0=male, 1=female), and whether individuals live in an urban or rural area (1=rural, 0=urban). The linear predictor from this very simple model is shown in equation 2.

Equation 2 $\ln\left(\frac{\hat{\mu}_i}{1-\hat{\mu}_i}\right) = \hat{\beta}_0 + \hat{\beta}_1 area_i + \hat{\beta}_2 sex_i + \hat{\beta}_3 age_i + \hat{\beta}_4 wealth_i$

We will obtain the predictive margin for each category of the area of residence. For the urban area predictive margin, we maintain adjustment covariates at their observed values but set area=0. Adjusted probabilities are obtained for each individual and then averaged over the whole sample:

Equation 3: $\hat{p}_i^{urban} = \frac{e^{\widehat{\beta}_0+\widehat{\beta}_2 sex_i+\widehat{\beta}_3 age_i+\widehat{\beta}_4 wealth_i}}{1+e^{\widehat{\beta}_0+\widehat{\beta}_2 sex_i+\widehat{\beta}_3 age_i+\widehat{\beta}_4 wealth_i}}$

Equation 4: $\hat{p}^{urban} = \frac{1}{\sum_i^n w_i}\sum_{i=1}^n w_i p_i^{urban}$ w_i: sampling design weight

The same procedure follows for the predictive margin of rural area:

$$\hat{p}^{rural} = \frac{1}{\sum_i^n w_i}\sum_{i=1}^n w_i \left(\frac{e^{\widehat{\beta}_0+\widehat{\beta}_1+\widehat{\beta}_2 area_i+\widehat{\beta}_3 age_i+\widehat{\beta}_4 wealth_i}}{1+e^{\widehat{\beta}_0+\widehat{\beta}_1+\widehat{\beta}_2 area_i+\widehat{\beta}_3 age_i+\widehat{\beta}_4 wealth_i}}\right)$$

Note that for each individual in the sample, their particular values of adjustment covariates are used to obtain their specific adjusted probability. For example, if an individual is male then sex=0 whereas for female individuals the sex covariate is maintained at sex=1. If instead predicted probabilities at the mean were calculated then the sex variable would be required to be substituted by its mean value (sex=0.52 for this example, 52% of the sample were female). Since no individual actually has a sex value of 0.52 we would be estimating in a point that definitely is not in the sample. As it was pointed out, predictive margins have the advantage of using actual covariate values for the covariate adjustment. Korn and Graubard highlight some other advantages of using predictive margins [1]. The following table shows covariate-adjusted probabilities for both methods applied to data from the 2012 Mexican National Health and Nutrition Survey along with unadjusted probabilities. Adjustment covariates were specified as in equation 2.

Table AF2-1. Area of residence predictive margins and predictions at the mean from a logistic regression model of body weight excess (BMI≥25).

Area of residence category	Predictive margins	Predictions at the mean of covariates	Unadjusted probabilities
Urban	0.728 ± 0.005	0.737 ± 0.005	0.734 ± 0.005
Rural	0.692 ± 0.008	0.701 ± 0.008	0.666 ± 0.008

n=35,851.

Predictive margins are interpreted as the averaged probability that would be observed if all subjects were at the category of interest (e.g. urban area). Therefore, predictive margins adjust probabilities for the distribution of covariates. They may be also calculated for given values of continuous covariates, for combinations of categorical covariates, or restricted to a subgroup of interest. The latter provide adjusted probabilities under the distribution of covariates observed in the subgroup of interest. We calculated predictive margins under the distribution of covariates observed in the whole sample.

References

1. Korn EL, Graubard BI. Analysis of Health Surveys. New York: Wiley 1999: 126-140
