# Supplementary material for: Time trends and sex differences in associations between socioeconomic status indicators and overweight-obesity in Mexico (2006–2012)
Source: BMC Public Health. 2015 Dec 16;15:1244. doi: 10.1186/s12889-015-2608-2 (PMC4682269; doi:10.1186/s12889-015-2608-2)
Supplement: Additional file 3: — Interaction tests with area of residence. (PDF 57 kb) [file 12889_2015_2608_MOESM3_ESM.pdf]

Additional File 3. Interaction tests with area of residence

We included interaction terms of area of residence [0=urban, 1=rural] with all covariates in our original models which already had all up three way interactions of survey year and sex with SES indicators. For each sex-year combination (men in 2006, men in 2012, women in 2006 and women in 2012), joint significance tests were performed for the group of parameters related to the covariate. For example, occupational status has one reference category and two indicator (0/1) variables of the rest of categories. In the reference group (e.g. men in 2006) we tested for the joint significance of these two terms with area of residence. For any of the other sex-year groups, the proper coefficients can be obtained as linear combinations of model coefficients or equivalently the group of interest (e.g. Women in 2012) can be set as reference category to facilitate implementation.

Table AF3-1. Joint significance tests for interactions with area of residence for a given sex and survey year category from the overweight plus obesity logistic regression model

	2006			2012		
	Statistic		P	Statistic		P
Men						
Wealth	F(2, 2800) =	0.11	0.894	**F(2, 2800) =**	**3.07**	**0.047**
Education	F(4, 2798) =	1.46	0.212	**F(4, 2798) =**	**2.10**	**0.079**
Marital status	F(2, 2800) =	1.68	0.188	F(2, 2800) =	0.80	0.452
Occupational status	F(3, 2799) =	1.10	0.346	F(3, 2799) =	0.21	0.893
Age	F(2, 2800) =	1.60	0.202	F(2, 2800) =	0.65	0.523
Region	F(2, 2800) =	1.58	0.206	F(2, 2800) =	1.25	0.287
Women						
Wealth	F(2, 2800) =	0.44	0.642	F(2, 2800) =	1.10	0.333
Education	F(4, 2798) =	1.83	0.120	F(4, 2798) =	0.93	0.444
Marital status	F(2, 2800) =	2.26	0.104	F(2, 2800) =	0.34	0.714
Occupational status	F(3, 2799) =	0.47	0.704	F(3, 2799) =	0.41	0.747
Age	F(2, 2800) =	3.31	0.037	F(2, 2800) =	0.02	0.981
Region	F(2, 2800) =	0.64	0.526	F(2, 2800) =	0.02	0.985

Table AF3-2. Joint significance tests for interactions with area of residence for a given sex and survey year category from the obesity logistic regression model

	2006		2012	
	Statistic	P	Statistic	P
Men				
Wealth	F(2, 2800) = 0.96	0.382	**F(2, 2800) = 2.71**	**0.067**
Education	F(4, 2798) = 0.40	0.807	F(4, 2798) = 1.47	0.209
Marital status	F(2, 2800) = 1.22	0.297	F(2, 2800) = 0.62	0.540
Occupational status	F(3, 2799) = 1.45	0.227	F(3, 2799) = 0.34	0.798
Age	F(2, 2800) = 1.41	0.244	F(2, 2800) = 0.64	0.527
Region	F(2, 2800) = 0.49	0.610	F(2, 2800) = 0.87	0.419
Women				
Wealth	F(2, 2800) = 1.84	0.159	F(2, 2800) = 0.47	0.626
Education	F(4, 2798) = 0.34	0.848	F(4, 2798) = 0.93	0.445
Marital status	**F(2, 2800) = 2.70**	**0.067**	F(2, 2800) = 0.24	0.786
Occupational status	F(3, 2799) = 0.99	0.394	F(3, 2799) = 0.25	0.858
Age	F(2, 2800) = 0.29	0.745	F(2, 2800) = 0.23	0.798
Region	F(2, 2800) = 0.18	0.834	F(2, 2800) = 1.03	0.359
