# Supplementary material for: Time trends and sex differences in associations between socioeconomic status indicators and overweight-obesity in Mexico (2006–2012)
Source: BMC Public Health. 2015 Dec 16;15:1244. doi: 10.1186/s12889-015-2608-2 (PMC4682269; doi:10.1186/s12889-015-2608-2)
Supplement: Additional file 4: — Wealth relationships with OWOB and obesity using equally spaced wealth categories. (PDF 93 kb) [file 12889_2015_2608_MOESM4_ESM.pdf]

Additional File 4. Wealth relationships with OWOB and obesity using equally spaced wealth categories

Our main models for OWOB and obesity were estimated with a categorical wealth variable instead of a continuous type indicator. Categories were defined to be equally spaced (except for the extremes) through the formula

$$Wealth\ category = round(7(wealth - min(wealth))/(max(wealth) - min(wealth))) + 1$$

Where the round function rounds the number to the nearest integer, $min(wealth)$ is the minimum or lowest value of the wealth indicator and $max(wealth)$ the maximum or highest value of the wealth indicator

Table AF4-1. Wealth categories and sample sizes

Wealth Category	Sample sizes				Wealth index	
	Men 2006	Men 2012	Women 2006	Women 2012	Min	Max
1	55	45	70	58	-3.859	-3.480
2	258	240	381	255	-3.439	-2.704
3	609	719	987	877	-2.696	-1.922
4	1,085	1,368	1,614	1,765	-1.920	-1.148
5	2,479	2,903	4,038	4,175	-1.147	-0.373
6	4,230	4,883	6,490	6,996	-0.373	0.398
7	3,137	3,927	4,517	5,328	0.402	1.168
8	667	1,055	841	1,257	1.206	1.563
Total	12520	15140	18938	20711	-3.859	1.563

Figure AF4-1. Wealth-OWOB relationship from a model with equally spaced wealth index categories

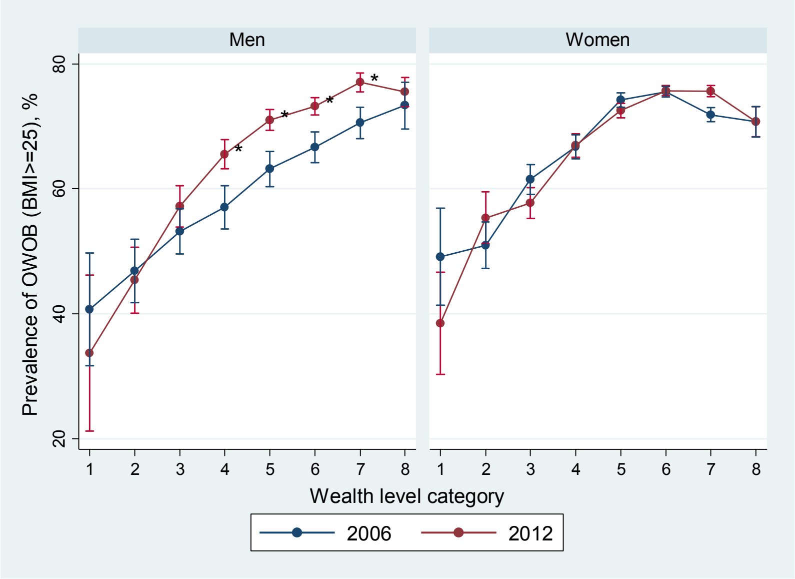

Error bars represent standard errors.*p<0.05 2012 vs 2006

Figure AF4-2. Wealth-obesity relationship from a model with equally spaced wealth index categories

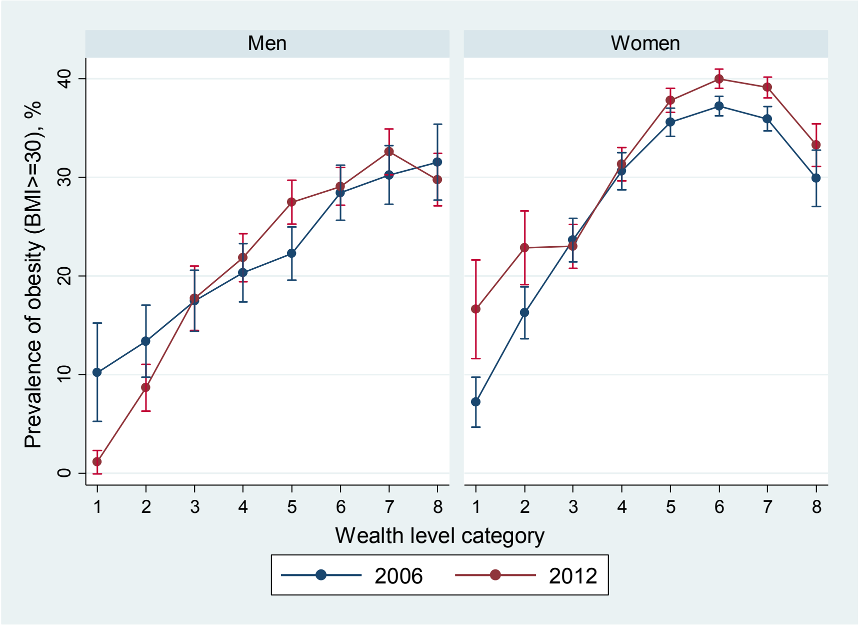

Error bars represent standard errors. No significant differences were found between 2006 and 2012.

In women at levels 6 and 7, p-values were near to the significance level (p=0.053 and p=0.051, respectively).
