# Supplementary material for: Time trends and sex differences in associations between socioeconomic status indicators and overweight-obesity in Mexico (2006–2012)
Source: BMC Public Health. 2015 Dec 16;15:1244. doi: 10.1186/s12889-015-2608-2 (PMC4682269; doi:10.1186/s12889-015-2608-2)
Supplement: Additional file 5: — Covariate adjusted slopes for the association between wealth and OWOB and obesity at given wealth levels, by sex and survey year. (PDF 87 kb) [file 12889_2015_2608_MOESM5_ESM.pdf]

Additional File 5. Covariate adjusted slopes for the association between wealth and OWOB and obesity, by sex and survey year

Covariate adjusted slopes were calculates as average marginal effects [1].

Table AF5-1. Covariate adjusted slopes for the association between wealth and OWOB (BMI≥25) at given wealth levels, by sex and survey year

Wealth Index (SD)	Men 2006	Men 2012	Men 2012 vs 2006	Women 2006	Women 2012	Women 2012 vs 2006
-3.5	7.7** ± 2.6	15.9*** ± 2.3	8.1* ± 3.5	20.5*** ± 2.2	20.4*** ± 2.0	-0.2 ± 3.0
-3.0	7.6** ± 2.3	14.8*** ± 2.4	7.2* ± 3.3	17.7*** ± 2.2	18.5*** ± 2.3	0.7 ± 3.2
-2.5	7.4*** ± 1.9	13.1*** ± 2.0	5.7* ± 2.8	14.1*** ± 1.8	15.3*** ± 2.0	1.3 ± 2.7
-2.0	7.1*** ± 1.5	11.0*** ± 1.6	3.9 ± 2.2	10.4*** ± 1.3	11.9*** ± 1.5	1.5 ± 2.0
-1.5	6.7*** ± 1.1	8.9*** ± 1.1	2.2 ± 1.6	7.2*** ± 0.9	8.7*** ± 1.0	1.5 ± 1.4
-1.0	6.4*** ± 0.9	7.0*** ± 0.8	0.6 ± 1.2	4.4*** ± 0.7	5.9*** ± 0.7	1.5 ± 1.0
-0.5	5.9*** ± 0.9	5.2*** ± 0.7	-0.7 ± 1.2	1.9** ± 0.6	3.5*** ± 0.6	1.6 ± 0.9
0.0	5.5*** ± 1.1	3.7*** ± 0.8	-1.8 ± 1.4	-0.4 ± 0.8	1.3 ± 0.7	1.7 ± 1.0
0.5	5.1*** ± 1.3	2.3* ± 1.0	-2.8 ± 1.7	-2.8** ± 1.0	-0.8 ± 1.0	2.0 ± 1.4
1.0	4.7** ± 1.6	1.0 ± 1.3	-3.6 ± 2.1	-5.4*** ± 1.5	-2.9* ± 1.3	2.4 ± 2.0
1.5	4.3* ± 1.8	-0.2 ± 1.7	-4.4 ± 2.4	-8.3*** ± 2.1	-5.3** ± 1.9	3.1 ± 2.8

§Wealth Index obtained by extracting the first principal component from household material characteristics, source of household water, electricity and possession of durable goods, and standardized with respect to 2006. Covariate adjusted slopes in percentage points ± standard errors are presented. Covariate adjusted slopes were obtained as average marginal effects. Adjustment covariates included age, age squared, attained education level, marital status, occupation status, country region and area of residence.
***P<0.001, **P<0.01, *P<0.05

Table AF5-1 Covariate adjusted slopes for the association between wealth and obesity (BMI≥30) at given wealth levels, by sex and survey year

Wealth Index§ (SD)	Men 2006	Men 2012	Men 2012 vs 2006	Women 2006	Women 2012	Women 2012 vs 2006
-3.5	4.3*** ± 0.8	5.5*** ± 0.6	1.2 ± 1.0	8.9*** ± 0.6	9.4*** ± 0.5	0.5 ± 0.7
-3.0	4.8*** ± 1.0	6.7*** ± 0.7	1.9 ± 1.2	10.5*** ± 0.7	10.8*** ± 0.7	0.3 ± 1.0
-2.5	5.1*** ± 1.1	7.4*** ± 0.9	2.3 ± 1.4	11.0*** ± 1.0	11.1*** ± 1.1	0.2 ± 1.5
-2.0	5.3*** ± 1.1	7.6*** ± 1.0	2.3 ± 1.5	10.2*** ± 1.1	10.3*** ± 1.2	0.1 ± 1.6
-1.5	5.3*** ± 1.0	7.3*** ± 1.0	1.9 ± 1.4	8.4*** ± 1.0	8.6*** ± 1.0	0.1 ± 1.4
-1.0	5.2*** ± 0.9	6.4*** ± 0.9	1.2 ± 1.2	6.0*** ± 0.8	6.2*** ± 0.8	0.2 ± 1.1
-0.5	4.9*** ± 0.8	5.0*** ± 0.8	0.1 ± 1.1	3.1*** ± 0.7	3.5*** ± 0.6	0.4 ± 1.0
0.0	4.5*** ± 1.0	3.4*** ± 0.9	-1.1 ± 1.3	0.1 ± 1.0	0.7 ± 0.8	0.6 ± 1.2
0.5	3.9** ± 1.5	1.5 ± 1.2	-2.4 ± 1.9	-2.9* ± 1.3	-2.2* ± 1.1	0.7 ± 1.7
1.0	3.3 ± 2.0	-0.4 ± 1.6	-3.7 ± 2.5	-5.8*** ± 1.6	-5.0*** ± 1.4	0.8 ± 2.1
1.5	2.6 ± 2.6	-2.3 ± 2.0	-4.8 ± 3.2	-8.3*** ± 1.7	-7.6*** ± 1.7	0.8 ± 2.4

§Wealth Index obtained by extracting the first principal component from household material characteristics, source of household water, electricity and possession of durable goods, and standardized with respect to 2006.

Covariate adjusted slopes in percentage points ± standard errors are presented. Covariate adjusted slopes were obtained as average marginal effects. Adjustment covariates included age, age squared, attained education level, marital status, occupation status, country region and area of residence.
***P<0.001, **P<0.01, *P<0.05

References

1. Cameron AC, Trivendi PK. *Microeconomics using Stata* 2 ed. Stata Press, College Station: TX 2009; 333-339 pp.
