# Supplementary figures and images for: Time trends and sex differences in associations between socioeconomic status indicators and overweight-obesity in Mexico (2006–2012)
Source: BMC Public Health. 2015 Dec 16;15:1244. doi: 10.1186/s12889-015-2608-2 (PMC4682269; doi:10.1186/s12889-015-2608-2)

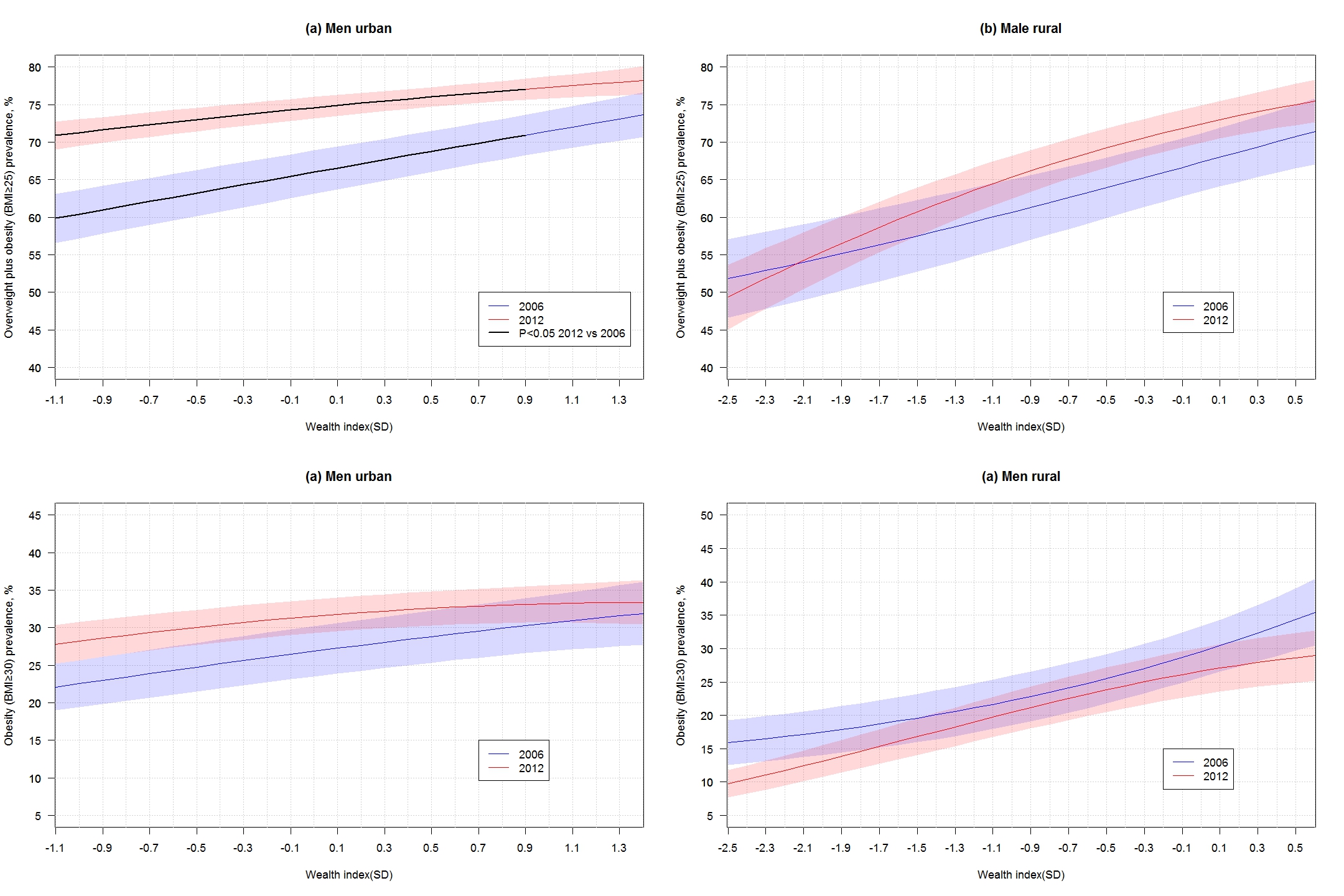

Supplement: Additional file 6: — Wealth associations for men, by area of residence. (JPEG 1072 kb) [file 12889_2015_2608_MOESM6_ESM.jpeg]
