# Supplementary material for: Time trends and sex differences in associations between socioeconomic status indicators and overweight-obesity in Mexico (2006–2012)
Source: BMC Public Health. 2015 Dec 16;15:1244. doi: 10.1186/s12889-015-2608-2 (PMC4682269; doi:10.1186/s12889-015-2608-2)
Supplement: Additional file 7: — Estimates from interactions with area of residence. (PDF 58 kb) [file 12889_2015_2608_MOESM7_ESM.pdf]

**Additional File 7. Estimates from interactions with area of residence**

**OWOB (BMI≥25) prevalences by survey year, level of education and area of residence for men**

| | Men Urban | | Men Rural | |
|---|---|---|---|---|
| | 2006 | 2012 | 2006 | 2012 |
| No education | $65.0^{a} \pm 4.2$ | $77.2^{ab*} \pm 3.1$ | $64.2^{a} \pm 5.1$ | $63.6^{a} \pm 3.9$ |
| Elementary school | $66.5^{a} \pm 2.9$ | $72.9^{a} \pm 1.8$ | $68.1^{ab} \pm 4.4$ | $67.9^{a} \pm 3.2$ |
| Middle school | $65.9^{a} \pm 3.1$ | $73.1^{ab*} \pm 1.8$ | $74.0^{b} \pm 4.2$ | $71.9^{b} \pm 3.1$ |
| High school | $67.3^{a} \pm 3.4$ | $76.8^{b*} \pm 1.9$ | $66.6^{ab} \pm 5.4$ | $74.5^{b} \pm 3.6$ |
| College/University | $66.5^{a} \pm 4.3$ | $77.0^{ab*} \pm 2.1$ | $58.8^{ab} \pm 8.7$ | $80.4^{b*} \pm 4.0$ |

Overweight plus obesity defined as BMI≥25
All estimates are covariate-adjusted prevalences ± standard errors obtained through predictive margins from a survey design-based multiple logistic regression model. Country region, area of residence, a wealth index and its squared term, age and age squared, marital status and occupational status were included in the model. All up to forth-way interactions of each adjustment covariate with survey year, sex and area of residence were also included in the model. Different letters indicate significant differences ($P<0.05$) between covariate categories in the same sex and survey year.*$P<0.05$ change from 2006 to 2012 within sex

**Obesity (BMI≥30) prevalences by survey year, marital status and area of residence for women**

| | Women Urban | | Women Rural | |
|---|---|---|---|---|
| | 2006 | 2012 | 2006 | 2012 |
| Single | $33.4^{a} \pm 1.9$ | $36.7^{a} \pm 1.6$ | $23.5^{a} \pm 3.4$ | $33.7^{a*} \pm 2.9$ |
| Married/cohabitating | $35.6^{a} \pm 1.1$ | $39.0^{a*} \pm 0.9$ | $33.3^{b} \pm 2.1$ | $35.6^{a} \pm 1.7$ |
| Widowed/separated | $36.5^{a} \pm 2.0$ | $38.7^{a} \pm 1.5$ | $28.3^{ab} \pm 3.0$ | $37.3^{a*} \pm 2.4$ |

Obesity defined as BMI≥30
All estimates are covariate-adjusted prevalences ± standard errors obtained through predictive margins from a survey design-based multiple logistic regression model. Country region, area of residence, a wealth index and its squared term, age and age squared, education level and occupational status were included in the model. All up to forth-way interactions of each adjustment covariate with survey year, sex and area of residence were also included in the model. Different letters indicate significant differences ($P<0.05$) between covariate categories in the same sex and survey year.*$P<0.05$ change from 2006 to 2012 within sex
