# Supplementary material for: Time trends and sex differences in associations between socioeconomic status indicators and overweight-obesity in Mexico (2006–2012)
Source: BMC Public Health. 2015 Dec 16;15:1244. doi: 10.1186/s12889-015-2608-2 (PMC4682269; doi:10.1186/s12889-015-2608-2)
Supplement: Additional file 8: — Comparison of models depending on the inclusion of wealth, education, or both as covariates. (PDF 59 kb) [file 12889_2015_2608_MOESM8_ESM.pdf]

Additional File 8. Comparison of models depending on the inclusion of wealth, education, or both as covariates

Table 1. Comparison of logistic regression models for overweight plus obesity that differ in whether education, wealth or both groups of variables were included as adjustment covariates.

	Model 1	Model 2	Model 3
Men 2006			
Wealth index	1.308*** ± 0.070		1.302*** ± 0.066
Wealth index squared	0.987 ± 0.023		0.990 ± 0.021
No education		1	1
Elementary school		1.372** ± 0.137	1.174 ± 0.125
Middle school		1.521*** ± 0.187	1.184 ± 0.157
High school		1.639*** ± 0.231	1.189 ± 0.183
College/University		1.692** ± 0.330	1.142 ± 0.234
Men 2012			
Wealth index	1.298*** ± 0.052		1.231*** ± 0.056
Wealth index squared	0.944** ± 0.020		0.93*** ± 0.020
No education		1	1
Elementary school		1.154 ± 0.134	0.978 ± 0.117
Middle school		1.335* ± 0.174	1.030 ± 0.141
High school		1.699*** ± 0.235	1.255 ± 0.185
College/University		1.839*** ± 0.285	1.299 ± 0.221
Women 2006			
Wealth index	0.88** ± 0.038		0.975 ± 0.043
Wealth index squared	0.845*** ± 0.017		0.873*** ± 0.017
No education		1	1
Elementary school		1.270** ± 0.115	1.115 ± 0.104
Middle school		1.096 ± 0.126	0.938 ± 0.112
High school		0.878 ± 0.101	0.785* ± 0.093
College/University		0.642** ± 0.095	0.602*** ± 0.093
Women 2012			
Wealth index	0.991 ± 0.036		1.079 ± 0.046
Wealth index squared	0.852*** ± 0.016		0.884*** ± 0.018
No education		1	1
Elementary school		1.671*** ± 0.152	1.452*** ± 0.136
Middle school		1.534*** ± 0.156	1.262* ± 0.135
High school		1.226 ± 0.144	0.999 ± 0.125
College/University		0.883 ± 0.105	0.740* ± 0.100

Coefficients are odds ratios ± standard error. *p<0.01; **p<0.05; ***p<0.001.
The three models included the following adjustment covariates: indicator variables of marital status, indicator variables of occupation status, age, age squared, indicator variables of country region and of area of residence. All up to three way interactions of each covariate with survey year and sex were also included. For brevity only coefficients related to wealth or education level are presented. Model 1 excluded the group of covariates of education level, model 2 excluded the wealth index and Model 3 corresponds to the model presented in the main results of this research.

Table 2. Comparison of logistic regression models for obesity that differ in whether education, wealth or both groups of variables were included as adjustment covariates.

	Model 1	Model 2	Model 3
Men 2006			
Wealth index	1.239*** ± 0.061		1.263*** ± 0.067
Wealth index squared	0.950 ± 0.025		0.963 ± 0.026
No education		1	1
Elementary school		1.484** ± 0.194	1.277 ± 0.169
Middle school		1.514** ± 0.229	1.205 ± 0.187
High school		1.582* ± 0.286	1.191 ± 0.227
College/University		1.493* ± 0.267	1.064 ± 0.200
Men 2012			
Wealth index	1.204*** ± 0.047		1.18*** ± 0.051
Wealth index squared	0.914*** ± 0.022		0.913*** ± 0.022
No education		1	1
Elementary school		1.215 ± 0.154	1.071 ± 0.138
Middle school		1.438** ± 0.189	1.183 ± 0.161
High school		1.465** ± 0.206	1.177 ± 0.169
College/University		1.486** ± 0.209	1.167 ± 0.175
Women 2006			
Wealth index	0.909** ± 0.034		1.004 ± 0.044
Wealth index squared	0.84*** ± 0.018		0.87*** ± 0.019
No education		1	1
Elementary school		1.178 ± 0.102	1.054 ± 0.093
Middle school		1.048 ± 0.114	0.927 ± 0.105
High school		0.775* ± 0.089	0.711** ± 0.090
College/University		0.615*** ± 0.080	0.587*** ± 0.087
Women 2012			
Wealth index	0.96 ± 0.030		1.029 ± 0.035
Wealth index squared	0.857*** ± 0.017		0.881*** ± 0.018
No education		1	1
Elementary school		1.357*** ± 0.112	1.234* ± 0.103
Middle school		1.142 ± 0.106	1.019 ± 0.097
High school		0.991 ± 0.107	0.892 ± 0.100
College/University		0.772* ± 0.093	0.725* ± 0.093

Coefficients are odds ratios ± standard error. *p<0.01; **p<0.05; ***p<0.001.
The three models included the following adjustment covariates: indicator variables of marital status, indicator variables of occupation status, age, age squared, indicator variables of country region and of area of residence. All up to three way interactions of each covariate with survey year and sex were also included. For brevity only coefficients related to wealth or education level are presented. Model 1 excluded the group of covariates of education level, model 2 excluded the wealth index and Model 3 corresponds to the model presented in the main results of this research.
